# Plasma Proteomics in IgA Nephropathy: From Circulating Biomarkers to Molecular Endotypes

**DOI:** 10.3390/cells15151373

**Published:** 2026-07-30

**Authors:** Charlotte Delrue, Stefania Marzocco, Rafael Noal Moresco, Marijn M. Speeckaert

**Affiliations:** 1 Department of Nephrology, Ghent University Hospital, 9000 Ghent, Belgium; charlotte.delrue@ugent.be; 2 Department of Pharmacy, University of Salerno, Via Giovanni Paolo II 132, 84084 Fisciano, Italy; smarzocco@unisa.it; 3 Graduate Program in Pharmaceutical Sciences, Center of Health Sciences, Federal University of Santa Maria, Santa Maria 97105-900, Brazil; rnmoresco@ufsm.br; 4 Research Foundation-Flanders (FWO), 1000 Brussels, Belgium

**Keywords:** IgA nephropathy, proteomics, molecular endotypes, precision medicine, complement activation, biomarkers, plasma proteomics, urinary proteomics, kidney tissue proteomics, mass spectrometry

## Abstract

IgA nephropathy (IgAN) is a primary glomerular disease with various clinical features, disease progression, and therapeutic responses that affects people worldwide. Currently, risk stratification depends primarily on clinical variables, together with the Oxford MEST-C classification, which indicates structural damage. However, these approaches only provide a limited explanation of the molecular mechanisms responsible for the disease. Recent proteomic discoveries have enabled researchers to characterize proteins not only in blood plasma and urine but also in kidney tissues, opening new avenues for understanding the underlying biology of IgAN. Our review addresses existing findings in plasma proteomic studies and, at the same time, brings into the picture developments in urinary and tissue proteomic profiling, thereby demonstrating that molecular profiling has been essential for further understanding of IgAN pathogenesis. Several research studies highlight complement system dysregulation, immune system overactivity, extracellular matrix remodeling, and metabolic disturbances as the leading factors linked to disease activity and progression. Even after many years in biomarker discovery, the development and clinical application of single plasma-based molecules as markers for disease detection remain challenging. Proteomic signatures based on the various processes involved in a single disease consistently outperform single protein identification in describing complexity and distinguishing molecular endotypes. We also present the current status of proteomic-based profiling methods, cross-linking proteomics with other data sources, and the remaining clinical application barriers. Through the development of this technology, proteomics has not only enabled the discovery of new biomarkers but also provided a framework for viewing IgAN as a heterogeneous disease comprising distinct molecular endotypes. Overall, current evidence indicates that proteomics is evolving from biomarker discovery toward molecular disease classification, with the potential to improve prognostication, guide mechanism-based therapeutic selection, and advance precision nephrology in IgAN.

## 1. Introduction

### 1.1. IgA Nephropathy: An Unmet Clinical Challenge

IgA nephropathy (IgAN) is the most common primary glomerular disease worldwide and remains a leading cause of chronic kidney disease (CKD) and kidney failure, particularly among young adults. Although its reported incidence varies widely across geographic regions, reflecting differences in genetic susceptibility, biopsy practices, and screening strategies, IgAN is the most common biopsy-proven primary glomerulonephritis in Europe and East Asia. It contributes substantially to the global burden of CKD [1,2].

The clinical presentation of IgAN is markedly heterogeneous [1,3]. For some patients, isolated microscopic hematuria may be the only symptom. Others may experience recurrent episodes of macroscopic hematuria after mucosal infections. Still others may present with asymptomatic proteinuria, nephrotic syndrome, rapidly progressive glomerulonephritis, or even advanced CKD [1]. Different clinical manifestations are now understood not as simple phases of a single condition but as signs of distinct biological events and the molecular mechanisms underlying them. For instance, a patient with only hematuria might be expected to have relatively mild signs of inflammation and scarring, whereas nephrotic syndrome often presents with more profound podocyte injury, complement activation, and glomerular membrane defects. Similarly, fulminant IgAN is marked by heavy immune involvement, with crescent formation, inflammatory cell influx, and activation of complement-mediated pathways. In addition, the development of CKD is increasingly linked to fibrosis and metabolic disturbance in tissues outside the kidney, along with remodeling of extracellular collagenous structures [4,5,6,7,8,9,10]. Similarly, the natural history can range from a quiet course with preserved kidney function for life to severe nephropathy requiring kidney replacement therapy within one or two decades [1,3]. This marked interindividual variability remains one of the greatest challenges in the management of IgAN, as disease progression cannot be reliably predicted at the time of diagnosis [3]. Approximately 30 to 40% of patients develop kidney failure within 20 years of diagnosis despite the contemporary standard of care [1,3].

Today, many management strategies rely primarily on clinical risk factors rather than directly assessing disease biology. The main factors that inform therapeutic decision-making are persistent proteinuria, blood pressure, a decline in estimated glomerular filtration rate (eGFR), and the extent of chronic histological changes. Supportive care measures such as renin-angiotensin system inhibition, blood pressure control, sodium restriction, and, more recently, sodium-glucose cotransporter-2 (SGLT2) inhibition have shown clinical benefit and dramatically reduce the risk of disease progression. Supportive care plays a vital role in the treatment of IgAN, and all efforts should be made to optimize it as much as possible before considering immunosuppressive or other targeted therapies. KDIGO recommendations, apart from dietary sodium restriction (<2 g/day), recommend maintaining a healthy body weight, smoking cessation, and regular physical exercise. They suggest optimizing blood pressure (target systolic blood pressure < 120 mmHg, when possible), achieving the highest level of renin-angiotensin system blockade tolerated, prescribing SGLT2 inhibitors to qualified patients, and providing statin treatment based on the individual’s cardiovascular risk profile [3,11]. These lifestyle changes significantly reduce proteinuria and help keep disease progression under control. In patients with persistent proteinuria despite supportive care at maximal doses, further treatment may be warranted to match the individual risk of disease progression. Patients with a targeted risk of disease progression, where progression is likely to occur sooner if not intervened, could be candidates for Nefecon treatment, i.e., targeted-release budesonide, a corticosteroid delivered to the distal ileum [12,13]. Targeted-release corticosteroids suppress mucosal IgA in the distal ileum and may be the most favored therapy in patients facing a higher risk of progression. In clinical trials, patients receiving targeted-release budesonide not only saw their proteinuria levels go down and their decline in kidney function slow (compared with placebo), but their corticosterone levels also did not go up. This means fewer problems from corticosterone, like weight gain. In the latest trial, sparsentan proved to be much better at lowering proteinuria than irbesartan [14].

Sparsentan acts by blocking not only the angiotensin II receptors but also the endothelin type A receptors. In this context, SGLT2 inhibitors, i.e., dapagliflozin and empagliflozin, have shown that in patients diagnosed with CKD, the risk of kidney disease progression as well as cardiovascular events was much lower. So these medications are considered fundamental therapy in most patients [3,11,15,16]. New mechanism agents targeting specific pathogenic pathways are changing the therapeutic landscape. Complement inhibitors like iptacopan, a specific blocker of factor B in the alternative complement pathway, have shown clinically noticeable reductions in proteinuria while maintaining kidney function in recent clinical trials [17].

However, many patients continue to develop irreversible nephron loss. In addition, immunosuppressive therapies have not consistently demonstrated benefit, and when they do, these benefits are often outweighed by serious adverse effects, underscoring the need for more accurate patient selection. Nevertheless, considerable interindividual variability in disease progression persists despite optimal supportive care, suggesting that conventional clinical risk factors alone do not adequately capture the underlying biological heterogeneity of IgAN and reinforcing the need for molecular biomarkers to guide individualized treatment. Similarly, the rapid proliferation of therapeutic agents targeting the mucosal immune system, A Proliferation-Inducing Ligand (APRIL) signaling, endothelin signaling, and various components of the complement cascade again underscores the need to identify patients most likely to benefit from mechanism-based interventions, rather than employing uniform treatment algorithms [3,11].

Major advances in understanding IgAN pathogenesis have transformed the disease from a histological diagnosis to a mechanistically defined immune-mediated disorder [18]. The currently accepted multi-hit model proposes that genetically susceptible individuals exhibit increased production of galactose-deficient IgA1 (Gd-IgA1), followed by the generation of anti-glycan autoantibodies, the formation of circulating immune complexes, mesangial deposition, and the activation of inflammatory and complement pathways, ultimately driving glomerular injury [1,18,19].

Despite these advances, the multi-hit hypothesis does not fully explain the extraordinary heterogeneity observed in clinical practice [4,18]. Patients with similar circulating concentrations of Gd-IgA1, comparable degrees of immune complex deposition, or nearly identical histopathological lesions often show markedly different rates of kidney function decline and variable responses to immunomodulatory therapy. Conversely, progressive fibrosis and loss of kidney function may occur despite apparently limited glomerular inflammation, suggesting that additional molecular processes govern disease progression [4]. These observations indicate that the current pathogenic model, although biologically compelling, remains incomplete and that important determinants of disease activity remain to be identified [4,18].

These limitations have significant implications for therapeutic development. The current generation of targeted therapies targets highly specific molecular pathways, including APRIL signaling, B-cell activation, endothelin signaling, and multiple components of the complement cascade. Their successful implementation will require biomarkers capable of identifying pathway activation in individual patients [3,11]. Histology alone is unlikely to provide this level of biological resolution [3]. Instead, there is an increasing need for molecular phenotyping strategies that complement traditional pathology by identifying biologically coherent patient subgroups and enabling mechanism-based therapeutic selection [11].

Proteomics, in particular, is a promising approach to addressing this unmet need. Because proteins are the primary functional agents in almost all biological processes, a detailed proteomic analysis may more directly reflect the evolving molecular environment of IgAN than genomic or transcriptomic analyses alone. By analyzing thousands of proteins in blood, urine, or tissue, proteomics can pinpoint altered pathways, uncover previously unknown mechanisms, characterize molecular endotypes, and identify biomarkers that not only enhance diagnosis but also help forecast and guide treatment strategies.

### 1.2. Limitations of Current Clinicopathological Classification

Although there have been significant advances in understanding and treating IgAN, predicting patients’ outcomes remains largely based on clinical factors and histopathological examination. Kidney biopsy remains the best way to diagnose the disease. In contrast, factors such as urine protein excretion, eGFR, blood pressure, and tissue description scores are major elements of current prognostic models [11,20]. These methods have greatly enhanced clinical management [11]. Still, they primarily measure the structural outcomes of the disease rather than the molecular mechanisms underlying disease onset, progression, and treatment response [20].

The introduction of the Oxford Classification marked a landmark achievement in renal pathology by replacing descriptive histological assessment with a standardized, reproducible scoring system. The original MEST score, later expanded to include crescents (MEST-C), identified five histopathological variables independently associated with renal outcome: mesangial hypercellularity (M), endocapillary hypercellularity (E), segmental glomerulosclerosis (S), tubular atrophy/interstitial fibrosis (T), and cellular or fibrocellular crescents (C) [20,21]. Numerous validation studies across diverse ethnic populations have confirmed the prognostic significance of these lesions, particularly the strong predictive value of tubulointerstitial fibrosis and tubular atrophy (T lesions), which consistently show the strongest association with long-term decline in renal function [20,22].

The success of the Oxford Classification should not obscure its fundamental limitations. Histopathological evaluation primarily provides a static morphological snapshot captured at a single time point within a dynamically changing disease process. This means that similar microscopic tissue changes might indicate quite different biological situations, influenced by disease duration, inflammation levels, compensatory mechanisms, or even prior medical treatments. However, individuals with distinct underlying molecular alterations may eventually develop histological changes that appear very similar, particularly in advanced fibrosis. Thus, morphology reflects the cumulative consequences of injury rather than its underlying biological drivers [4].

This distinction is becoming increasingly important as the therapeutic landscape of IgAN evolves. Novel agents targeting specific pathogenic pathways, including B-cell maturation, APRIL signaling, endothelin signaling, and complement activation, assume that patients differ by dominant molecular mechanisms rather than histological phenotype alone. However, conventional pathology cannot identify activation of these pathways. Neither mesangial hypercellularity nor segmental sclerosis provides clues about complement pathway activation, immune cell composition, extracellular matrix remodeling, metabolic reprogramming, or intracellular signaling networks. As a result, histological classification provides only a very limited biological context and is, of course, inadequate for implementing precision medicine based on the underlying mechanisms.

Another inherent limitation of kidney biopsy is sampling variability. A diagnostic biopsy samples less than 0.002% of total renal tissue, yet IgAN exhibits considerable spatial heterogeneity, both within individual glomeruli and across cortical regions. Lesions such as focal segmental sclerosis, crescents, or localized inflammatory infiltrates may therefore be missed, depending on the biopsy core obtained. Although current recommendations advocate obtaining at least eight glomeruli for reliable pathological assessment, sampling error remains unavoidable, particularly in early disease or focal lesions [17,20].

Interobserver variability presents an additional challenge. Although the Oxford Classification substantially improved reproducibility compared with earlier descriptive systems, agreement remains variable across individual pathological lesions. Tubular atrophy/interstitial fibrosis generally shows excellent reproducibility, whereas mesangial hypercellularity, especially endocapillary hypercellularity, shows only moderate interobserver agreement, reflecting the subjective nature of lesion interpretation [17,23]. Crescents present further complexity because their biological significance appears to depend on concomitant immunosuppressive therapy, disease stage, and chronicity, reducing their independent prognostic value in treated populations [20].

Perhaps the greatest conceptual limitation of the current clinicopathological classification is its inability to explain the remarkable clinical heterogeneity observed among patients with apparently similar disease [4,24]. Individuals with identical MEST-C scores frequently experience profoundly different clinical trajectories. While some patients with extensive mesangial proliferation remain asymptomatic for many years, others with very mild histological changes experience rapid deterioration that leads to kidney failure [24]. Similarly, patients with comparable levels of proteinuria and similar eGFR at the time of diagnosis may respond completely differently to corticosteroids, targeted-release budesonide, and complement inhibitors [4]. This variability clearly indicates that clinicopathological features alone do not fully reveal the biological heterogeneity of IgAN [4,24].

The International IgA Nephropathy Prediction Tool represents a major advance by integrating clinical variables with Oxford pathology to estimate an individualized risk of renal function decline [24]. External validation studies have consistently shown that prognostic performance is superior to that of clinical or pathological parameters alone. However, although the tool generally demonstrates good discrimination, its calibration varies across ethnic and geographic populations, indicating that local recalibration or validation may be required before routine clinical implementation in specific populations. Nevertheless, even this model predicts outcomes without directly measuring disease biology. The variables in the prediction tool remain surrogate markers of structural damage and kidney function rather than molecular pathway activation. Consequently, the model predicts who is likely to progress but offers little insight into why progression occurs or which therapeutic pathway to target.

The growing recognition of complement dysregulation highlights this limitation particularly well. Genetic studies have identified susceptibility variants in complement factor H-related proteins (CFHR), while immunohistochemical and proteomic studies consistently demonstrate glomerular deposition of C3, C4d, properdin, factor H, mannose-binding lectin, and terminal complement components. Importantly, patients with similar Oxford scores may show markedly different complement activation profiles, suggesting biologically distinct disease endotypes that conventional histopathology alone cannot distinguish [5,6]. Similar observations have emerged regarding inflammatory signaling, extracellular matrix remodeling, and metabolic adaptation, underscoring that morphology alone cannot capture the molecular diversity of IgAN.

When viewed through the lens of precision nephrology, these shortcomings become even more apparent. Precision medicine calls for biomarkers that can recognize active disease mechanisms, forecast treatment outcomes, track disease status over time, and even detect molecular changes before structural damage becomes permanent. Histopathology is not even close to meeting these standards. Kidney biopsy is invasive, unsuitable for serial monitoring, and primarily evaluates irreversible structural lesions rather than dynamic biological activity. Consequently, the current clinicopathological paradigm remains largely reactive, detecting injury only after substantial tissue damage has already occurred rather than identifying the molecular perturbations responsible for disease progression.

Taken together, these observations indicate that contemporary classification systems have reached an important conceptual ceiling. Histology remains essential for diagnosing and evaluating chronic structural injuries. However, it offers only limited insight into the functional molecular networks that control disease progression.

### 1.3. From the Multi-Hit Hypothesis to Molecular Heterogeneity

The multi-hit hypothesis has profoundly changed the way we think about IgAN: the disease is no longer viewed as a vaguely characterized histopathological entity but as a truly mechanism-based, immune-mediated disorder. Over the past two decades, extensive genetic, biochemical, immunological, and experimental data have revealed a sequential pathogenetic pathway. This model holds that genetically predisposed individuals produce higher levels of Gd-IgA1, which serves as the target for antiglycan autoantibodies, leading to the formation of circulating immune complexes that are then deposited in the glomerular mesangium, triggering inflammatory and complement-mediated damage [7,18,19]. This view of the disease has significantly shaped both fundamental scientific investigations and drug discovery. It has provided a sound biological basis for new therapies targeting mucosal immunity, B-cell activation, APRIL signaling, and complement pathways.

The first pathogenic hit involves aberrant O-glycosylation of the hinge region of IgA1, leading to the formation of Gd-IgA1. Multiple genome-wide association studies have demonstrated that susceptibility to abnormal IgA1 glycosylation is substantially under genetic control, involving genes regulating mucosal immunity, glycosyltransferase activity, antigen presentation, and complement regulation [25]. However, elevated circulating Gd-IgA1 concentrations alone are insufficient to cause disease. Elevated serum Gd-IgA1 levels are also observed in clinically unaffected relatives of patients with IgAN, indicating that abnormal glycosylation is a necessary but not sufficient pathogenic event [25,26].

The second pathogenic hit is the production of IgG and IgA autoantibodies that specifically target galactose-deficient hinge-region glycans. These autoantibodies help form circulating high-molecular-weight immune complexes that have a greater capacity to cause nephritis [7,18]. Experimental data reveal that these complexes induce mesangial cells to proliferate, release cytokines, produce extracellular matrix, and activate complement [7]. As a result, they link systemic immune imbalance to glomerular damage [7,18].

Later, immune complexes deposited in the mesangium trigger a cascade of inflammatory responses [5,6,7,18]. This includes mesangial cell activation and the secretion of interleukin-6 (IL-6), tumor necrosis factor-alpha (TNF-α), monocyte chemoattractant protein-1 (MCP-1), transforming growth factor-beta (TGF-β), platelet-derived growth factor, and several chemokines that not only attract leukocytes but also promote extracellular matrix accumulation [7]. At the same time, activation of the alternative and lectin complement pathways exacerbates tissue damage by generating C3 activation fragments, C5a-mediated inflammatory signaling, and the formation of the membrane attack complex (C5b-9) [5,6]. The complement system, once thought to be a mere byproduct of immune complex deposition, has now emerged as a major driver of ongoing renal injury, as supported by genetic, histopathological, and proteomic findings [4,5,6].

However, despite its considerable explanatory power, the multi-hit hypothesis falls short of accounting for the extraordinary biological and clinical heterogeneity observed in IgAN. In fact, patients with similar serum Gd-IgA1 levels may exhibit strikingly different clinical presentations, lesion severity, and progression to renal failure over time. Similarly, considerable overlap exists between healthy individuals and patients in circulating Gd-IgA1 concentrations and antiglycan autoantibody levels, limiting the utility of these measures as standalone biomarkers [7,18]. These observations indicate that additional molecular determinants modulate disease susceptibility and progression beyond the classical pathogenic sequence.

One conceptual limitation of the multi-hit model is its inherently linear representation of disease pathogenesis. Although it is highly effective as a visualization system, it portrays disease development as a sequential process. In contrast, up-to-date systems biology reveals a far more complex network of interacting molecular pathways. Immune activation, complement dysregulation, endothelial injury, podocyte dysfunction, extracellular matrix remodeling, mitochondrial dysfunction, metabolic reprogramming, oxidative stress, and tubulointerstitial inflammation are interlinked through highly connected signaling networks that evolve with the disorder, rather than being isolated events happening one after another. In fact, this means that the pathological feature of IgAN is increasingly understood as a dynamic molecular network rather than a simple chain of separate pathogenic hits.

Recent multi-omics studies combining genomics, transcriptomics, spatial transcriptomics, and proteomics have made this complexity even more evident. These studies have consistently reported significant molecular diversity among patients with the same clinicopathological features. Single-cell and spatial transcriptomic studies have revealed disease-specific alterations in mesangial cells, podocytes, endothelial cells, tubular epithelial cells, infiltrating macrophages, T cells, and B cells, and have also demonstrated that the spatial organization and interactions of these cell populations vary considerably between patients, even among those with similar clinicopathological findings [8]. However, spatial transcriptomic methods have shown that inflammatory signaling can vary significantly across regions of the same biopsy sample, highlighting the spatial complexity of immune responses in the kidney.

Proteomic studies have extended these observations by demonstrating marked heterogeneity in complement activation, extracellular matrix remodeling, immune signaling, and metabolism among patients with similar clinicopathological features. These findings support the concept that IgAN comprises multiple molecular endotypes [9,10,27]. Importantly, this molecular heterogeneity also provides a biological explanation for the diverse clinical presentations of IgAN. Rather than being a manifestation of the same underlying disease, these findings, including asymptomatic hematuria, recurrent episodes of gross hematuria, both non-nephrotic and nephrotic proteinuria, rapidly progressive IgAN, and CKD with slower progression, could each arise through varied complement activation, different levels of immune dysregulation, various cellular interactions, different patterns of kidney structural remodeling, varying adaptations to metabolic challenges, and distinct layouts of inflammatory responses within the parenchymal kidney cells. By correlating clinical signs and lab findings with plasma, urine, and kidney tissue proteomics and applying spatial transcriptomics, we could identify more precisely molecular endotypes with clinical relevance and improve our prognostication of disease progression and patient response to treatments [7,24,25,26,27,28].

**Figure 1:**
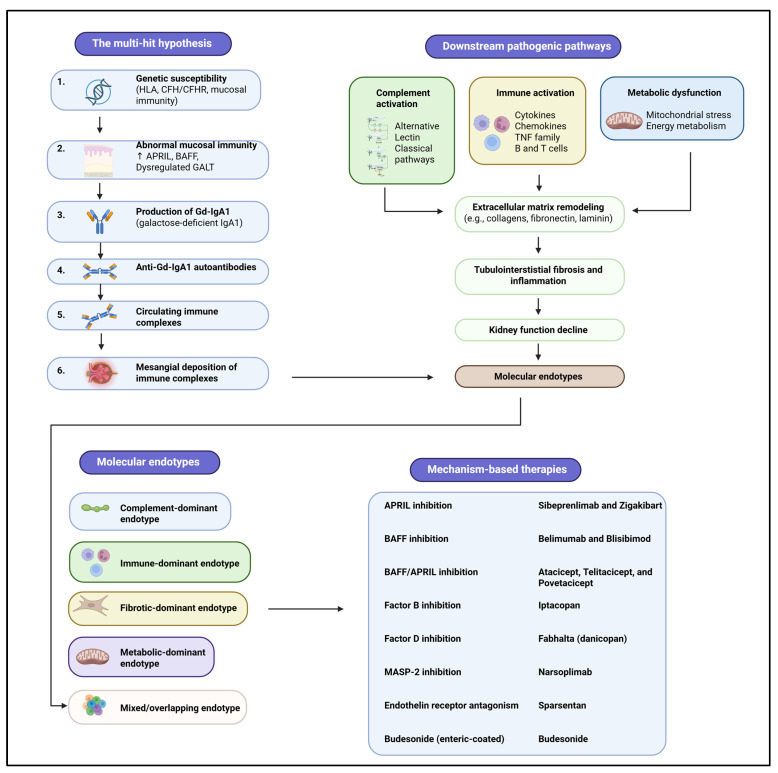
Conceptual framework linking the classical multi-hit hypothesis with downstream pathogenic pathways, molecular endotypes, and mechanism-based therapies in IgA nephropathy. Abbreviations: APRIL, A Proliferation-Inducing Ligand; BAFF, B-cell Activating Factor; CFH, complement factor H; CFHR, complement factor H-related protein; GALT, gut-associated lymphoid tissue; Gd-IgA1, galactose-deficient immunoglobulin A1; MASP-2, mannose-binding lectin-associated serine protease 2.

Recognizing molecular heterogeneity has important therapeutic implications. Current treatment strategies increasingly target specific pathogenic pathways, including APRIL signaling, complement activation, endothelin signaling, and inflammatory cytokine networks [4,9]. However, activation of these pathways is unlikely to be uniform across patients, making a single therapeutic approach unlikely to benefit all individuals equally. Instead, treatment response is expected to depend on the dominant molecular mechanisms at play in each patient [4]. Consequently, the classical multi-hit hypothesis should be regarded not as the final model of IgAN pathogenesis, but as the biological foundation for a more comprehensive framework that incorporates downstream molecular pathways, distinct molecular endotypes, and mechanism-based therapeutic strategies [4,9]. This conceptual framework is summarized in Figure 1. Recognizing this molecular heterogeneity has important implications for biomarker development. If patients with similar clinical and histopathological characteristics differ in their dominant biological pathways, traditional clinicopathological classification alone is unlikely to provide sufficient resolution for precision medicine. Consequently, technologies that capture pathway-level biological activity have become increasingly important, providing the principal rationale for contemporary proteomic investigations in IgAN [4,9,28].

### 1.4. Why Proteomics?

The growing recognition of molecular heterogeneity in IgAN has fundamentally reshaped the requirements for disease classification. Traditional clinicopathological variables remain indispensable for diagnosis and risk stratification but offer limited insight into the biological mechanisms driving disease progression. Similarly, advances in genomics and transcriptomics have substantially improved our understanding of disease susceptibility and cellular responses, but they do not fully capture the functional molecular processes underlying tissue injury. Consequently, there is an increasing need for technologies capable of interrogating the dynamic biological pathways that ultimately determine disease phenotype, therapeutic responsiveness, and long-term outcome. Among the currently available omics platforms, proteomics occupies a unique position because proteins constitute the principal functional mediators of virtually all cellular processes and therefore represent the closest molecular approximation to phenotype [28].

In contrast to the genome, which remains largely the same throughout the lifespan, and the transcriptome, which reveals a cell’s transcriptional capacity, the proteome is highly dynamic and changes in response to signaling environments, immune activation, metabolic shifts, therapeutics, and disease progression. Beyond gene transcription, protein levels are also influenced by mRNA stability, translational efficiency, intracellular trafficking, secretion, proteolytic degradation, and numerous post-translational modifications. As a result, mRNA levels often show only a weak correlation with protein abundance, particularly in complex inflammatory diseases that are tightly regulated at the post-transcriptional level [29,30]. This lack of correspondence between mRNA and protein levels is particularly important in IgAN, where complement system activation, immune complex production, extracellular matrix remodeling, and cytokine signaling are primarily regulated at the protein level rather than solely by transcription.

Proteomics may offer a major advantage for directly measuring the molecular components underlying disease phenotypes. Almost all significant pathogenic mechanisms in IgAN, including complement activation, immunoglobulin glycosylation, matrix remodeling, oxidative stress, mitochondrial dysfunction, endothelial activation, podocyte injury, and tubulointerstitial fibrosis, are mediated by proteins. In addition, many biological processes depend on post-translational modifications that cannot be inferred from transcriptomic data. Post-translational modifications, exemplified by the proteolytic cleavage of complement proteins, phosphorylation of intracellular signaling molecules, glycosylation of IgA1, oxidation of mitochondrial proteins, and cross-linking of the extracellular matrix, are molecular events that modulate protein function without necessarily changing gene expression [28]. That is why proteomics delivers direct mechanistic insights that complement genomic and transcriptomic analyses.

An equally important advantage of proteomics is its ability to interrogate multiple biological compartments simultaneously [31,32]. Plasma proteomics captures systemic immune activation, complement dysregulation, coagulation pathways, and inflammatory signaling [31]. Urinary proteomics reflects dysfunction of the glomerular filtration barrier, tubular injury, extracellular matrix turnover, and intrarenal inflammation, while providing a noninvasive source of molecular information suitable for longitudinal monitoring [31,32]. Tissue proteomics, particularly when combined with laser capture microdissection or spatial proteomic technologies, enables direct characterization of compartment-specific molecular alterations within glomeruli, tubules, interstitium, and renal vasculature [32]. Together, these complementary biological matrices provide a multiscale representation of disease biology, spanning systemic immune dysregulation to local cellular injury [31,32].

This integrated approach is particularly relevant because the clinical phenotypes of IgAN are unlikely to stem from identical biological mechanisms. Combining plasma, urinary, and tissue proteomics with spatially resolved molecular profiling offers an opportunity to link clinical presentation to the dominant molecular pathways and cellular interactions active within individual patients, thereby supporting mechanism-based classification and precision therapeutic selection [25,28,31,32].

Technological advances over the past decade have transformed clinical proteomics from a relatively low-throughput exploratory discipline into a mature platform for large-scale molecular phenotyping. Modern high-resolution liquid chromatography tandem mass spectrometry (LC-MS/MS), particularly data-independent acquisition (DIA), now enables reproducible quantification of several thousand proteins from limited clinical samples and substantially reduces missing data compared with traditional data-dependent acquisition methods [33]. In parallel, affinity-based platforms such as Olink proximity extension assays and SomaScan aptamer technology enable simultaneous measurement of thousands of circulating proteins across large patient cohorts, thereby facilitating biomarker discovery and molecular epidemiology on an unprecedented scale [34,35]. Importantly, these technologies should be viewed as complementary rather than competing approaches, with mass spectrometry enabling unbiased protein discovery and affinity-based assays enabling high-throughput validation in large clinical populations. The principal proteomic platforms currently used in IgAN research, together with their analytical characteristics, strengths, limitations, and major applications, are summarized in Table 1.

Beyond biomarker discovery, contemporary proteomics increasingly integrates pathway enrichment, protein interaction networks, and machine learning to identify coordinated biological programs rather than isolated proteins [9,27].

Alongside these major benefits, there are still several key drawbacks [28,31]. Protein levels in plasma differ by more than ten billionfold, making it highly challenging to detect very low-concentration biomarkers [28]. Pre-analytic factors, such as how the sample was collected, how it was stored, the number of times it was frozen and thawed, methods for protein removal, and approaches to standardization, still contribute significantly to technical variation. There are also substantial disparities between mass spectrometry and affinity-based methods in analytical sensitivity, protein coverage, reproducibility, and quantification, making direct comparison of results across studies difficult [28,31]. Last but not least, many proteomic studies published to date have been cross-sectional, relatively small in sample size, and lacking independent external validation, which restricts their clinical application for the time being [31].

Nevertheless, these limitations should be viewed within the broader context of a rapidly evolving field. Advances in data-independent acquisition, single-cell proteomics, spatial proteomics, extracellular vesicle proteomics, artificial intelligence, and multimodal data integration continue to expand both the analytical depth and biological resolution of clinical proteomics. Rather than serving solely as a source of diagnostic biomarkers, proteomics is increasingly emerging as a framework for redefining disease in terms of functional molecular pathways.

Accordingly, the following sections review how plasma proteomics has advanced from biomarker discovery toward molecular endotyping and its potential role in precision nephrology. Importantly, although proteomic investigations have been conducted in plasma, urine, and kidney tissue, the primary focus of this review is plasma proteomics. We specifically examine how circulating proteomic signatures contribute to identifying dysregulated molecular pathways, biomarker discovery, prognostic stratification, prediction of therapeutic response, and the definition of molecular endotypes in IgAN [9,28]. Rather than providing an exhaustive overview of all proteomic applications in nephrology, this review critically evaluates the strengths, limitations, and clinical relevance of plasma proteomic studies and discusses their integration with complementary urinary and tissue proteomic approaches where relevant [9,28,31]. Finally, we synthesize findings across plasma, urinary, and tissue proteomic studies to assess whether reproducible molecular signatures converge into biologically meaningful disease endotypes. Particular attention is paid to complement-dominant, immune-inflammatory, fibrotic/remodeling, metabolic, and mixed molecular endotypes, which may ultimately provide a framework for precision medicine in IgAN [9,27,36].

Three key questions guide this review: (1) which circulating proteomic signatures reproducibly characterize IgAN; (2) whether plasma proteomics provides biological and prognostic information beyond established clinicopathological tools, such as the Oxford MEST-C classification and the International IgA Nephropathy Prediction Tool; and (3) whether plasma proteomic profiling can identify clinically actionable molecular endotypes that support precision medicine strategies [9,24,28]. By addressing these questions, we aim to determine whether plasma proteomics is evolving beyond biomarker discovery toward a framework for molecular disease classification and for mechanism-based therapeutic stratification in IgAN [9,28].

Although this review focuses primarily on plasma proteomics, relevant findings from urinary and kidney tissue proteomics are discussed when they provide complementary biological context or independent validation of circulating proteomic signatures. These additional biological matrices are not reviewed comprehensively but are included to facilitate interpretation of plasma-derived molecular pathways and their relationship to intrarenal disease mechanisms.

## 2. Plasma Proteomics

### 2.1. Discovery Studies

Using plasma proteomics to study IgAN has evolved significantly over the past twenty years, driven by advances in proteomic techniques and a deeper understanding of disease pathogenesis. Plasma proteomics has transformed our understanding of IgAN by shifting the field from descriptive biomarker discovery toward systems-level characterization of disease biology. Rather than identifying isolated biomarkers, proteomic studies consistently highlight coordinated alterations in complement activation, immune and inflammatory signaling, extracellular matrix remodeling, and metabolism, supporting the concept that IgAN comprises biologically distinct molecular endotypes. Initially, research focused on generating hypotheses and identifying blood biomarkers to distinguish IgAN patients from healthy individuals or from those with other kidney diseases. Current studies increasingly employ comprehensive plasma proteomic profiling to identify dysregulated biological pathways, define molecular endotypes, and discover therapeutic targets. This shift reflects a broader transition from merely identifying biomarkers to conducting system-wide molecular phenotyping, a development in clinical proteomics that has had an impact across different fields of medicine [28]. The major plasma proteomic discovery studies that have shaped this evolution, together with their study populations, analytical platforms, and principal findings, are summarized in Table 2.

Initial plasma proteomic studies relied predominantly on two-dimensional gel electrophoresis (2-DE), surface-enhanced laser desorption/ionization time-of-flight mass spectrometry (SELDI-TOF-MS), and matrix-assisted laser desorption/ionization time-of-flight (MALDI-TOF) platforms. Although technically innovative at the time, these approaches suffered from limited proteome coverage, poor reproducibility, narrow dynamic range, and inadequate detection of low-abundance proteins. Most studies identified only several dozen to a few hundred proteins, with abundant plasma proteins such as albumin, immunoglobulins, fibrinogen, transferrin, and haptoglobin dominating the spectra. Consequently, many potentially relevant inflammatory mediators, cytokines, complement proteins, and signaling molecules remained below the analytical detection limit. In addition, considerable methodological heterogeneity in sample preparation, protein depletion, normalization strategies, and statistical analyses hindered comparisons between studies and limited reproducibility across independent cohorts [28,31].

Despite these technical limitations, the earliest discovery studies were instrumental in showing that the circulating proteome of patients with IgAN differs substantially from that of healthy individuals. Repeatedly observed differential expression of proteins involved in innate immunity, acute-phase responses, lipid metabolism, coagulation, oxidative stress, and extracellular matrix turnover suggested that IgAN is characterized by systemic biological alterations extending beyond the kidney. Importantly, several independent studies identified abnormalities in complement-related proteins, providing early proteomic evidence supporting histopathologic and genetic associations and indicating that complement dysregulation is a central pathogenic mechanism in IgAN. Although individual protein candidates varied considerably across studies, convergence on common biological pathways represented an important conceptual advance.

The introduction of high-resolution LC-MS/MS represented a major technological advance [31,33]. Compared with gel-based methods, LC-MS/MS greatly increased proteome coverage, enabling the simultaneous quantification of several thousand circulating proteins and improving the reproducibility and sensitivity of analysis. More recently, data-independent acquisition (DIA) has further improved quantitative accuracy by fragmenting all detectable peptide ions rather than selecting only the most abundant precursors for analysis [33]. As a result, current plasma proteomic studies routinely quantify between 2000 and 5000 different proteins from very small plasma samples, providing researchers with much more information about disease biology than older discovery methods [31,33]. Representative discovery studies illustrating this technological evolution, from early gel-based proteomics to contemporary LC-MS/MS- and DIA-based approaches, are summarized in Table 2.

The application of these next-generation technologies has fundamentally altered the objectives of plasma proteomic research in IgAN. Rather than identifying isolated diagnostic biomarkers, investigators increasingly employ unbiased proteomic profiling to reconstruct dysregulated molecular pathways. For instance, integrated plasma proteomic and metabolomic analyses using DIA-MS revealed that activation of complement and immune signaling pathways, together with alterations in amino acid and energy metabolism, were linked to changes released by this disease. More importantly, pathway-level analyses were much more informative than individual protein measurements, identifying coordinated biological programs rather than single molecular changes. Machine-learning methods produced biomarker panels that include proteins such as protein kinase cAMP-dependent type II regulatory subunit alpha (PRKAR2A), interleukin-6 signal transducer (IL6ST), and Son of Sevenless homolog 1 (SOS1), which successfully differentiated patients with IgAN from healthy controls with high diagnostic accuracy; however, external validation remains very limited.

Another important development is the emergence of affinity-based proteomic platforms, including proximity extension assays (PEA; Olink) and aptamer-based technologies (SomaScan). These platforms provide highly sensitive quantification of predefined protein panels and are particularly well suited for large epidemiological studies because they require only small sample volumes while maintaining excellent analytical reproducibility. Although their protein coverage is limited to predefined targets and they lack the discovery potential of untargeted mass spectrometry, affinity-based assays have greatly facilitated the validation of candidate biomarkers identified in discovery cohorts. Increasingly, unbiased LC-MS/MS discovery followed by targeted validation using Olink or enzyme-linked immunosorbent assay (ELISA) has become the preferred workflow for translational biomarker development.

Beyond differential expression analysis, systems biology approaches are increasingly being integrated into contemporary plasma proteomics. Methods such as network analysis, weighted protein co-expression analysis, pathway enrichment, protein–protein interaction mapping, and machine learning help identify coordinated biological programs rather than individual biomarkers. These methods have revealed that the pathways most often altered in these studies include complement activation, innate immunity, extracellular matrix remodeling, coagulation, lipid metabolism, and mitochondrial function. Thus, alterations in circulating proteomics may reflect integrated biological networks rather than independent protein abnormalities. These findings have shifted the emphasis from biomarker discovery to elucidating molecular endotypes that may account for the striking clinical heterogeneity observed among patients with similar clinicopathological attributes.

Nevertheless, significant limitations remain. First, most discovery studies have relied on relatively small patient cohorts, some with fewer than 100 individuals, resulting in limited statistical power and an increased likelihood of false positives. Second, validating findings across different populations remains rare. Third, variations in sample handling, protein removal, analytical platforms, normalization methods, and bioinformatics pipelines make studies unpredictable and incomparable. Furthermore, plasma proteins vary widely with age, kidney function, systemic inflammation, medications, and cardiovascular disease, raising genuine concerns about disease specificity. Several proteins identified as potential biomarkers for IgAN, including those related to inflammation (cytokines and acute-phase proteins), are elevated in other chronic kidney diseases as well. That means it is important to separate the general effects of decreased kidney function from the disease’s specific molecular changes [37].

Another conceptual limitation is that most discovery studies remain cross-sectional. As a result, the identified proteins are associated with established disease rather than with those proteins causally involved in disease initiation or progression. Differences in protein abundance do not necessarily indicate biological significance, and circulating proteins may reflect responses to kidney injury rather than primary pathogenic mechanisms. Therefore, combining genetics, transcriptomics, spatial proteomics, and longitudinal clinical phenotyping will be important for establishing causal relationships and for differentiating mechanistically relevant biomarkers from epiphenomena.

### 2.2. Complement Signatures

Complement dysregulation is among the most consistently identified circulating proteomic signatures in IgAN [38]. Plasma and serum proteomic studies have moved the field beyond measuring single complement proteins toward pathway-level interpretation [36]. In an integrated LC-MS/MS-based proteomic and metabolomic study, complement and immune-related pathways were among the most prominently altered in IgAN [27], supporting the view that circulating proteomic changes reflect coordinated biological programs rather than isolated candidate biomarkers [36].

The strongest genetic and proteomic evidence converges on the complement factor H (CFH)-CFH-related protein axis. Genetic studies have shown that variants in the CFH/CFHR region influence susceptibility to IgAN, consistent with altered regulation of alternative pathway amplification [39]. More recent multi-omics work using proteomic, genetic, and transcriptomic triangulation identified complement factor H as a genetically supported protective factor in IgAN, suggesting that more efficient CFH-mediated regulation may reduce disease susceptibility [40]. This is important because it shifts CFH from being merely an associated circulating protein to a biologically plausible causal regulator.

Complement factor H-related proteins are equally important. Circulating CFHR1 and CFHR5 have been associated with IgAN disease activity, supporting the concept that dysregulated competition between CFH and CFHR proteins may promote activation of the alternative pathway [41]. Recent studies of circulating IgA-containing immune complexes have further refined this concept. Proteomic analysis of serum IgA1-containing immune complexes identified increased levels of complement proteins and apolipoproteins in patients with IgAN, supporting the involvement of the alternative and lectin pathways rather than dominance of the classical pathway [42]. A separate study found that IgA-containing immune complexes contained elevated levels of C1q, CFHR1, and properdin; CFHR1 within immune complexes was higher in patients selected for immunosuppressive therapy and decreased after treatment, correlating with remission [43]. These observations suggest that complement proteins bound to immune complexes may be more informative about disease than total plasma concentrations.

The lectin pathway also appears relevant. Proteomic analysis of IgA1-containing immune complexes supports the involvement of lectin pathway proteins, and histological studies have linked lectin pathway activation, especially C4d deposition, to more severe disease and worse outcomes [42]. This matters therapeutically because emerging complement-targeted agents inhibit complement activation. Complement proteomics may help identify patients in whom complement activation is a dominant pathogenic program, although this remains a hypothesis rather than an established clinical tool.

The main limitation is specificity. Systemic inflammation, liver synthesis, eGFR, proteinuria, infection, and treatment exposure strongly influence circulating complement proteins. Moreover, total plasma abundance of complement proteins does not necessarily reflect pathway activation. Activation fragments, immune-complex-bound complement proteins, and tissue deposition may be more informative than intact circulating proteins. Platform differences also matter: LC-MS/MS, DIA-MS, Olink, SomaScan, and immunoassays do not measure identical molecular entities and should not be treated as interchangeable.

### 2.3. Immune Activation

Plasma proteomics is shedding more light on IgAN. IgAN is a systemic, immune-mediated disorder rather than a disease confined to the kidney. Deposition of mesangial IgA-containing immune complexes in the kidney is the hallmark lesion. However, circulating protein profiles reveal involvement of a broader range of innate immune activation, B-cell and plasma-cell biology, TNF-superfamily signaling, chemokine networks, and monocyte–macrophage pathways. This aligns with the multi-hit model, which posits that mucosal immune dysregulation, the production of galactose-deficient IgA1, the formation of anti-glycan autoantibodies, and the generation of immune complexes occur before their deposition in the kidney and the subsequent inflammatory injury [7,18,44].

The TNF receptor pathway has emerged as another reproducible inflammatory axis. Kobayashi et al. measured serum inflammatory proteins in newly diagnosed IgAN and disease-control cohorts and found that TNF receptor-related proteins, particularly tumor necrosis factor receptor (TNF-R)1, TNF-R2, TNF-R3, TNF-R7, and TNF-R27, were elevated in IgAN and associated with the severity of tubulointerstitial lesions and subsequent decline in kidney function [45]. The inclusion of membranous nephropathy, minimal change disease, and lupus nephritis as disease controls strengthens the study design by partially addressing disease specificity. However, the finding that membranous nephropathy and lupus nephritis also showed elevations in several TNF receptor-related proteins indicates that these markers are not specific to IgAN. Rather, they likely identify a broader inflammatory kidney injury phenotype.

Chemokine signaling is another prominent theme in plasma proteomics. CCL2, CXCL1, and CXCL12 have been repeatedly implicated in IgAN across transcriptomic, proteomic, and experimental studies, consistent with the recruitment of monocytes, macrophages, neutrophils, and lymphocytes, as well as stromal immune interactions [9]. This is particularly relevant because tubulointerstitial inflammation and macrophage infiltration are among the strongest biological correlates of progression. Nevertheless, chemokines are highly context-dependent and are increased in many inflammatory and fibrotic kidney diseases. Their value may therefore lie less in diagnosis and more in defining an inflammatory endotype, especially when combined with markers of complement, extracellular matrix, and tubular injury.

The activation of B cells and plasma cells represents distinct immune axes with direct therapeutic relevance. B-cell Activating Factor (BAFF) and APRIL are cytokines of the TNF superfamily that, through signaling via B-cell Activating Factor Receptor (BAFF-R), Transmembrane Activator and CAML Interactor (TACI), and B-cell Maturation Antigen (BCMA), regulate B-cell survival, class-switch recombination, plasma-cell differentiation, and IgA production. In IgAN, these signaling pathways are mechanistically attractive and may support the production of galactose-deficient IgA1 and anti-glycan autoantibodies, the latter of which directly cause immune complex formation. This rationale has been supported by both experimental and clinical studies: BAFF-transgenic mice develop IgA-dominant immune abnormalities, and APRIL blockade decreases IgA, galactose-deficient IgA1, immune complex formation, proteinuria, and glomerular immune deposition in both preclinical and clinical settings [44].

From a proteomic perspective, the biology of BAFF/APRIL underscores an important distinction between inflammatory biomarkers and actionable immune pathways. Measuring circulating BAFF, APRIL, BCMA, TACI, or related TNF-superfamily proteins may provide insight into upstream mucosal and B-cell activation, but these proteins should not be viewed as isolated biomarkers. Their clinical relevance depends on whether they identify patients with active immune complex formation who are more likely to respond to APRIL or to dual BAFF/APRIL blockade. Current trials of sibeprenlimab, zigakibart, atacicept, telitacicept, and povetacicept support the therapeutic importance of this pathway, but predictive proteomic biomarkers for response have not yet been established [44].

A critical limitation of immune-activation signatures is their biological nonspecificity. eGFR, proteinuria, infection, obesity, cardiovascular disease, age, medication exposure, and systemic inflammation influence TNF receptors, chemokines, cytokines, and acute-phase proteins. This is particularly problematic in plasma, where many proteins originate from the liver, vascular endothelium, circulating leukocytes, lymphoid tissue, or inflamed extrarenal compartments rather than the kidney itself. Thus, plasma immune signatures must be interpreted as systemic inflammatory readouts unless directly linked to kidney tissue, urinary proteins, immune-complex composition, or clinical outcomes.

Another limitation is that many plasma immune studies are cross-sectional. They can identify associations with proteinuria, eGFR, Oxford lesions, or future decline, but they rarely establish causality. Inflammatory proteins may drive injury, mediate compensatory responses, or result from reduced kidney function. Future studies should therefore include serial sampling, disease controls matched for eGFR and proteinuria, paired kidney tissue, and predefined clinical endpoints. The most useful designs will be embedded in therapeutic trials, where plasma immune proteins can be tested as pharmacodynamic markers or predictors of response.

### 2.4. Prediction of Progression

Predicting disease progression could be the clinical application of plasma proteomics in IgAN that has the greatest impact. At the moment, doctors make prognostic judgments primarily based on proteinuria, eGFR, blood pressure, treatment history, and the Oxford MEST-C classification. These variables, first and foremost, indicate the extent of structural damage rather than the fundamental molecular pathways underlying disease progression. Plasma proteomics allows detection of blood-based signals of complement system overactivity, immune system problems, endothelial damage, tissue matrix changes, and metabolism-related disturbances, thereby refining risk prediction beyond the usual clinical indicators. Even so, the research is still in the early stages, with most studies being pilot and not validated by other researchers. Kobayashi et al. reported that multiple circulating inflammatory proteins, particularly TNF receptor-related proteins, including TNF-R1, TNF-R2, TNF-R3, TNF-R7, and TNF-R27, were elevated in IgAN and were associated with pathological lesions and a decline in kidney function [45]. This study is notable for linking circulating proteins to both histological injury and longitudinal decline, which is essential for progression biology. Its critical limitation is specificity: TNF receptor proteins are also associated with progression in other kidney diseases, especially diabetic kidney disease and inflammatory glomerular disorders, so they may represent a general progressive kidney injury phenotype rather than an IgAN-specific pathway [46].

The CFH-CFHR axis, alternative pathway activation, and immune-complex-associated complement proteins are mechanistically linked to IgAN and may help identify patients at higher risk of progression. Genetic and proteomic evidence supports CFH as a protective regulator, whereas CFHR proteins may promote alternative-pathway amplification by competing with CFH-mediated regulation [36]. From a prognostic standpoint, the key question is not whether complement activation is present, but whether a measurable circulating complement profile independently predicts future decline, independent of proteinuria, eGFR, and MEST-C lesions. That standard has not yet been consistently met across large prospective plasma proteomic cohorts. Therefore, complement signatures are mechanistically compelling but still require prospective validation as predictive biomarkers.

Integrated plasma proteomics and metabolomics have added another layer to the biology of progression. Zhang et al. used LC-MS/MS-based plasma proteomics and metabolomics to identify candidate biomarkers and altered pathways in IgAN, showing enrichment of immune, complement, and metabolic processes [27]. Such studies are valuable because progressive IgAN is unlikely to be explained by a single pathway. Instead, worsening kidney function probably reflects interactions among immune activation, complement amplification, metabolic stress, endothelial injury, and fibrosis. A key limitation is that many studies compare IgAN with healthy controls rather than with disease controls matched for eGFR and proteinuria, thereby inflating diagnostic performance and complicating prognostic interpretation.

For plasma proteomics to become clinically useful for predicting progression, several methodological standards are essential. First, models must be tested against established predictors, particularly baseline eGFR, proteinuria over time, and the International IgAN Prediction Tool. Second, studies must use clinically meaningful endpoints, such as a 30% or 40% decline in eGFR, kidney failure, or a sustained eGFR slope, rather than cross-sectional disease severity. Third, models require external validation in independent, multiethnic cohorts. Fourth, disease controls are essential because many plasma proteins associated with progression in IgAN are also altered in lupus nephritis, membranous nephropathy, diabetic kidney disease, and advanced CKD. Finally, biomarker panels should be locked before validation to reduce overfitting.

### 2.5. Why Have Most Plasma Biomarkers Failed Clinical Translation?

Despite more than twenty-two years of plasma proteomic studies in IgAN, no plasma protein biomarker has been used in daily clinical practice. Many biomarkers have been linked to diagnosis, disease activity, prognosis, and response to therapy. Only a few of these candidates have been independently validated across different patient populations. None has demonstrated analytical validity, clinical validity, or clinical utility to the extent that would warrant a role as a complementary or substitutive method for current prognostic tools. This discrepancy between laboratory findings and their utilization in the clinical realm, the so-called translation gap, seems to result from the complexity of the biology at stake, differences in analytical methods, and a lack of well-standardized methods overall.

One of the principal limitations is the marked biological heterogeneity of IgAN. Rather than a single disease entity, IgAN encompasses multiple molecular endotypes characterized by varying degrees of complement activation, mucosal immune dysregulation, inflammatory signaling, extracellular matrix remodeling, and metabolic adaptation.

A second major problem is that many circulating proteins have limited discriminatory power for disease. Complement proteins, acute-phase reactants, cytokines, chemokines, extracellular matrix proteins, and markers of endothelial activation change in IgAN as well as in diabetic kidney disease, lupus nephritis, anti-neutrophil cytoplasmic antibody (ANCA)-associated vasculitis, focal segmental glomerulosclerosis, and several non-renal inflammatory conditions. Moreover, blood protein levels are strongly affected by age, sex, kidney function, proteinuria, obesity, cardiovascular disease, infection, and drug treatment. So, identifying proteins that truly capture the essence of IgAN pathophysiology, rather than those that reflect systemic inflammation or renal clearance failure, remains challenging.

Technical variability further limits reproducibility. Plasma continues to be recognized as one of the hardest biological matrices to analyze because it contains a very wide range of protein concentrations. Also, variations in how samples are collected, prepared, and analyzed, as well as in the bioinformatics methods used, add substantially to variation between studies, frequently causing differences in the sets of proteins considered to be the ones mainly responsible for a given pathway-level convergence. Such methodological constraints are also a major problem in clinical translation. Almost all of these studies remain essentially cross-sectional; they rely on relatively small, single-center cohorts, and external validation or longitudinal follow-up is almost always missing. As a result, many of the biomarkers reported in these studies are simply reflections of the disease state as it existed, not predictors of disease progression. Inadequate adjustment for significant confounding factors, such as eGFR, proteinuria, treatment exposure, and systemic inflammation, greatly restricts the ability to establish causality interpretations. Finally, analytical validity alone is insufficient for clinical implementation. Candidate biomarkers must demonstrate reproducibility across analytical platforms, laboratories, and patient populations and provide incremental prognostic value beyond established clinical models, such as the International IgA Nephropathy Prediction Tool and the Oxford MEST-C classification. Equally important, biomarker-guided therapeutic decisions must ultimately improve patient outcomes in prospective interventional studies. At present, this level of evidence is lacking for virtually all proposed plasma biomarkers in IgAN.

Taken together, these limitations suggest that the greatest future contribution of plasma proteomics lies not in identifying individual biomarkers, but in defining robust molecular signatures that improve risk stratification and support precision medicine.

### 2.6. Prediction of Treatment Response

Predicting treatment response is the least mature yet most clinically important application of plasma proteomics in IgAN. Current therapeutic decisions remain largely based on proteinuria, eGFR, blood pressure, histological chronicity, and toxicity risk, rather than direct evidence that a specific pathogenic pathway is active in an individual patient. This is increasingly problematic as IgAN therapy shifts from nonspecific immunosuppression to pathway-directed interventions, including APRIL and BAFF inhibition, endothelin receptor antagonism, targeted-release budesonide, and complement inhibition. Plasma proteomics could therefore serve as a companion strategy to identify dominant molecular programs and match patients to mechanism-based therapy.

At present, however, no validated plasma proteomic test predicts treatment response in IgAN. Most of the available evidence is exploratory, observational, or pharmacodynamic, and few proteomic markers have been prospectively tested for treatment interactions in randomized trials. This distinction is critical: a biomarker associated with progression is not necessarily predictive of response to a specific therapy. Prognostic and predictive biomarkers should be distinguished at this stage to differentiate disease outcomes with or without treatment (prognostic biomarkers) from treatment response (predictive biomarkers). Prognostic biomarkers are associated with disease outcomes irrespective of treatment. They identify patients at higher risk of disease progression. Predictive biomarkers indicate which patients would derive greater benefit from a specific therapy. Thus, a biomarker correlated with disease progression alone is not indicative of a treatment effect.

The strongest rationale currently comes from the APRIL-BAFF axis. APRIL and BAFF regulate various aspects of B-cell function, including survival, plasma cell differentiation, class-switch recombination, and IgA production, through signaling via BAFF-R, TACI, and BCMA. Since the synthesis of galactose-deficient IgA1 and anti-glycan autoantibodies is the initial pathogenic step in IgAN, plasma levels of APRIL, BAFF, and other TNF-superfamily proteins can serve as effective biological indicators of drug response and as predictors for therapeutic agents that inhibit this pathway. Current evidence supports a role for BAFF and APRIL in the generation and survival of plasma cells that produce Gd-IgA1 and anti-Gd-IgA1 autoantibodies. However, their utility as predictive biomarkers for selecting patients most likely to benefit from BAFF/APRIL-targeted therapy remains unproven [44].

APRIL inhibition provides the clearest example. In the phase 2 sibeprenlimab trial, treatment reduced proteinuria more than placebo, supporting the therapeutic relevance of upstream APRIL blockade in IgAN [47]. In later phase 3 interim data, sibeprenlimab reduced APRIL and pathogenic Gd-IgA1 concentrations at week 48, suggesting that these proteins may serve as pharmacodynamic markers of pathway inhibition rather than as established baseline predictors of response [48]. Thus, APRIL and Gd-IgA1 should be interpreted as mechanistic readouts of target engagement, rather than validated tools for selecting patients before treatment.

Dual BAFF/APRIL blockade is also highly relevant. Available evidence suggests that BAFF/APRIL antagonists can reduce proteinuria and circulating Gd-IgA1, but the evidence base remains limited and has not yet established which baseline proteomic profiles identify likely responders [49]. This is a key gap. If BAFF/APRIL inhibition is most effective in patients with active upstream IgA production, future trials should test whether baseline levels of APRIL, BAFF, BCMA, TACI, immunoglobulin-related proteins, Gd-IgA1, or immune-complex-associated proteins modify treatment effect.

Complement-directed therapy presents a second major opportunity for plasma proteomics. Complement activation is a major pathogenic pathway in IgAN, and emerging therapies target factors B and D, C3, C5, and components of the lectin pathway [50]. In principle, plasma complement proteomics could distinguish patients with dominant activation of the alternative or lectin pathways from those whose disease is driven mainly by immune complex formation, inflammation, or established fibrosis. In practice, this remains unvalidated. Baseline complement signatures have not yet been shown, in prospective studies, to function as validated predictive biomarkers of response to a specific complement inhibitor in IgAN. A promising refinement is to measure complement proteins within circulating IgA-containing immune complexes rather than in unfractionated plasma. Tsuji et al. reported that IgA-containing immune complexes in IgAN showed increased levels of C1q, CFHR1, and properdin; CFHR1 abundance was higher in patients selected for immunosuppressive therapy and decreased after treatment, correlating with remission [43]. This suggests that immune-complex-associated complement proteins may be more closely linked to pathogenic activity than total plasma concentrations. If replicated, this approach could be more informative than conventional plasma complement measurement.

On the other hand, plasma proteomics is progressing more slowly in its efforts to address corticosteroids and other immunosuppressive therapies. These classes of drugs simultaneously activate multiple immune and metabolic pathways, making a single predictive signature unlikely. Plasma proteomics might even help identify transient inflammatory phases versus permanent fibrotic lesions. Nevertheless, this idea requires serial sampling, disease-control cohorts, and trial-embedded validation.

Several methodological standards are essential before plasma proteomics can guide therapy. Predictive biomarkers must be tested for treatment interaction in randomized trials, not merely associated with outcome. Studies need baseline and longitudinal sampling to separate predictors from pharmacodynamic changes. Disease activity must be distinguished from CKD burden, because many circulating inflammatory and complement proteins are affected by eGFR, proteinuria, infection, systemic inflammation, and treatment exposure. Finally, biomarker panels should be locked before external validation.

Overall, plasma proteomics has strong potential for predicting treatment response in IgAN, but the field is not yet ready for clinical use. The most promising directions are APRIL/BAFF pathway monitoring, immune-complex-associated complement profiling, and longitudinal proteomic assessment during targeted therapy. Its near-term value is likely to be trial enrichment and pharmacodynamic monitoring; its long-term value may be assignment of patients to complement-dominant, APRIL/BAFF-dominant, inflammatory, or fibrotic endotypes for precision therapy.

## 3. Integration of Plasma Proteomics with Other Molecular Compartments

### 3.1. Why Plasma Alone Is Insufficient 

Although plasma proteomics provides valuable information on systemic biological processes, urinary and kidney tissue proteomics provide complementary information on intrarenal molecular alterations and compartment-specific disease mechanisms. Urinary proteomics enables non-invasive longitudinal monitoring, whereas tissue proteomics directly characterizes molecular changes within specific renal compartments. The following sections summarize these complementary findings before integrating the evidence across all biological matrices in Section 3.4.

### 3.2. Urinary Proteomics: A Window into Intrarenal Injury

Compared with plasma, urine represents a unique molecular window into ongoing renal damage. While the circulating proteome primarily reflects systemic immune activation and host responses, the urinary proteome is produced primarily by the kidney and urinary tract. It comprises proteins and endogenous peptides released via glomerular filtration, tubular secretion, extracellular vesicles, apoptotic cells, and extracellular matrix turnover. Because of this, urinary proteomics is generally considered more closely aligned with intrarenal biological processes than plasma proteomics and has become one of the most advanced applications of proteomics in nephrology [51].

Plasma and urine differ in one important respect: the type of biological information they contain. Plasma primarily reveals the body’s initial pathological alterations, including immune dysregulation, complement activation, and systemic inflammation. Urine, on the other hand, shows later changes, such as tissue damage, extracellular matrix remodeling, and nephron destruction. These differences and compatibilities make urine proteomics highly promising for detecting disease activity and predicting prognosis. Another point in favor of urine proteomics is that, unlike a kidney biopsy, which is quite invasive, urine samples are noninvasive and can be collected repeatedly, allowing monitoring of disease progression and treatment response. Given its features, urinary proteomics is well-suited to serial molecular phenotyping, which is, after all, an important aspect of precision nephrology.

How capillary electrophoresis combined with mass spectrometry (CE-MS) has been a key driver in bringing urinary proteomics to the forefront of clinical technology is an excellent illustration of technological progress. CE-MS is well-suited for trace analysis of urinary peptides, including collagen fragments and low-molecular-weight proteins, which are poorly represented by traditional immunoassays. Over the past 20 years, such urinary peptide patterns have been developed into several disease-specific classifiers that have undergone thorough analytical and clinical validation and have been used to create large urinary peptide databases [51].

One of the major milestones has been the creation of the urinary peptide classifier IgAN237. It is a group of 237 urinary peptides that, when combined, reflect the molecular profile of IgAN. IgAN237 does not restrict us to only a few biomarkers but also captures various pathophysiological mechanisms, including extracellular matrix turnover, collagen breakdown, inflammation, and tubular damage. This combination enables a comprehensive systems-level portrayal of the disease. The results of the first multicenter study indicated that IgAN237 could predict a slow decline in kidney function better than traditional clinical parameters and demonstrated significant prognostic improvement over proteinuria and baseline eGFR. In the first multicenter study, incorporation of IgAN237 into a clinical prediction model significantly improved discrimination of patients with progressive kidney function decline, increasing the area under the receiver operating characteristic curve (AUC) from 0.72 (95% CI 0.64–0.81) for clinical parameters alone to 0.89 (95% CI 0.83–0.95) after inclusion of the urinary classifier, while also providing prognostic improvement beyond baseline proteinuria and eGFR [52].

Subsequent longitudinal studies further strengthened these observations. Peters and colleagues demonstrated that IgAN237 remains prognostically informative throughout the disease course, not only at the time of kidney biopsy. Importantly, serial measurements tracked disease progression and reflected temporal changes in molecular activity, suggesting that urinary proteomics may be useful for repeated risk assessment and disease monitoring. This dynamic behavior represents a major advantage over histopathological classification, which provides only a single time-point assessment and cannot capture evolving biological processes or longitudinal changes in disease activity [53].

Beyond forecast classifiers, urinary proteomics has revealed crucial aspects of the molecular machinery underlying IgAN evolution. Many independent investigations have identified the same modifications in collagen fragments, matrix proteins, inflammatory factors, and complement components. A decline in urinary collagen fragments is typically interpreted as a sign of reduced extracellular matrix degradation and consequent development of renal fibrosis, whereas an increase in tubular proteins indicates ongoing tubular damage. These findings support the idea that urinary peptide profiles reflect the dynamics of tissue restructuring and are not limited to changes in glomerular permeability alone [54].

Recent advances in high-resolution LC-MS/MS have expanded urinary proteomics beyond peptide profiling to the comprehensive characterization of the urinary proteome. In particular, several investigators have demonstrated enrichment of complement-related proteins in the urine of patients with IgAN. Wang and colleagues identified differential urinary abundance of multiple complement proteins and reported significant associations with proteinuria, Oxford MEST-C lesions, and renal function. Pathway enrichment analyses demonstrated activation of both the alternative and terminal complement pathways, providing further evidence that complement dysregulation is reflected not only in plasma and kidney tissue but also in urine [55].

Extracellular vesicles in urine are paving the way for a new and invigorated area of research. Podocytes, tubular epithelial cells, and immune cells release exosomes and microvesicles containing proteins, lipids, and nucleic acids that mirror the physiological state of their cells of origin. Because lipid membranes envelop extracellular vesicles, they might preserve biologically informative proteins that are usually degraded in whole urine. Although investigations of IgAN remain sparse, studies of the protein content of urinary extracellular vesicles have identified proteins associated with podocyte injury, cytoskeletal organization, inflammation, and fibrosis. This indicates great potential for future biomarker identification.

Urinary proteomics also has several major limitations despite breakthroughs. For one, protein expression in urine is affected by fluid intake, urine concentration, time of day, drug use, and sample handling. Therefore, standardization of urine collection, processing, and normalization methods remains essential to achieve reproducibility of results across centers. Additionally, urinary protein levels depend not only on proteinuria but also on disease-specific molecular changes, further complicating interpretation. As with plasma proteomics, variations in analytical platforms, bioinformatics pipelines, and statistical methods further hinder direct comparisons between studies. Finally, although classifiers such as IgAN237 have undergone external validation, prospective clinical trials demonstrating that urinary proteomics improves therapeutic decision-making remain scarce.

Overall, urinary proteomics is currently the most mature proteomic application in IgAN. Compared with plasma, it provides greater insight into ongoing intrarenal injury, tissue remodeling, and disease progression, while retaining the practical advantage of repeated, noninvasive sampling. Nevertheless, plasma and urine should not be regarded as competing biological matrices. Rather, they provide complementary information: plasma reflects systemic immune dysregulation, and urine captures downstream renal injury. Their integration, together with kidney tissue proteomics and other omics technologies, is likely to provide the most comprehensive molecular characterization of IgAN and to form the basis for future molecular endotyping and precision nephrology [52].

### 3.3. Kidney Tissue Proteomics: Defining the Molecular Architecture of IgA Nephropathy

Currently, kidney biopsy remains the most reliable method for confirming IgAN. Immunofluorescence microscopy can highlight glomerular IgA deposits, a defining feature of IgAN. A kidney biopsy not only confirms the presence of IgAN but also helps assess the extent of the disease by evaluating glomerular immune-complex deposition, histopathological lesions, and chronic structural damage. With the Oxford MEST-C classification, histopathological standardization and risk stratification have become more precise by quantifying mesangial hypercellularity, endocapillary hypercellularity, segmental glomerulosclerosis, tubular atrophy/interstitial fibrosis, and crescents [20]. Still, traditional histopathology is primarily descriptive and provides little insight into the molecular mechanisms underlying disease initiation, progression, or response to therapy. We see that patients with very similar MEST-C scores may have very different clinical courses, highlighting that histological lesions may be the endpoint of multiple biological processes rather than a single disease mechanism [24].

Kidney tissue proteomics is a promising method for linking morphology and molecular biology. While plasma and urine provide only indirect data on renal immune activation or injury, tissue proteomics yields molecular profiling of a diseased kidney. In this way, it reveals biological processes specific to the various kidney compartments—glomeruli, tubules, and tubulointerstitium and, because of this, connects molecular changes with histopathological lesions and patient outcome [56]. However, kidney tissue proteomics remains technically challenging because of the limited amount of biopsy material, the need for specialized tissue processing and MS workflows, and the marked spatial heterogeneity of renal tissue. Most studies have been based on relatively small patient cohorts, and, as a result, statistical power has been limited, and independent verification of the presented molecular signatures has been difficult. These technical and methodological limitations are worth considering when interpreting current results and, at the same time, point to the need for further investigation in larger, standardized, multicenter studies. The introduction of LCM and high-resolution LC-MS/MS has revolutionized renal proteomics. LCM enables the selective isolation of single glomeruli or tubulointerstitial compartments from formalin-fixed or frozen biopsy tissues, preventing contamination from adjacent tissues and enabling molecular profiling of each compartment. Compared with whole-biopsy proteomics, this technique greatly enhances biological resolution by pinpointing proteins related to glomerular inflammation, podocyte damage, or interstitial fibrosis. Major studies have shown that proteomic profiling of laser-microdissected glomeruli can effectively distinguish glomerular diseases based on their molecular fingerprints, thereby supporting the use of tissue proteomics as a complement to routine histopathology [56].

The application of these techniques to IgAN has consistently demonstrated that complement activation is a dominant molecular signature in affected glomeruli. Studies examining cellular protein composition have revealed elevated levels of several complement components, including C3, C5, C9, properdin, and complement factor H-related proteins. These data strongly indicate that the alternative complement pathway is continuously activated in the renal microenvironment [15,36]. The results from these studies are very similar to those from plasma proteomics and genetic investigations of the CFH-CFHR locus, which together show complementary dysregulation as a key pathogenic mechanism present at both systemic and tissue levels.

Beyond complement activation, tissue proteomics has highlighted profound alterations in extracellular matrix (ECM) remodeling. Progressive IgAN shows increased levels of collagens, laminins, fibronectin, and other extracellular matrix proteins, whereas the levels of proteins involved in the physiological degradation of the matrix are decreased. These molecular changes closely resemble the onset of glomerulosclerosis and tubulointerstitial fibrosis and are strongly linked to decreased kidney function. Proteomic analysis, then again, indicates that active extracellular matrix remodeling may occur before visible histological fibrosis, suggesting that molecular changes might help identify patients at risk of irreversible structural damage even before abnormalities become apparent in conventional histopathology [15].

Recent studies have also revealed that diseased kidneys undergo major metabolic changes. Proteomic studies show decreased levels of proteins that regulate mitochondrial oxidative phosphorylation, fatty acid oxidation, and cellular energy metabolism, mostly in the tubulointerstitial compartment. These results align with growing evidence that mitochondrial dysfunction leads to tubular injury and fibrosis in various chronic kidney diseases. In IgAN, dysfunctional mitochondrial metabolism appears to be directly linked to inflammatory processes and extracellular matrix accumulation, suggesting that a metabolic disorder may be a significant molecular driver of disease progression [16].

Inflammatory signaling is another prominent feature of renal tissue proteomics. More proteins involved in innate immune activation, leukocyte recruitment, antigen presentation, and cytokine signaling have been identified in the glomerular and tubulointerstitial compartments. Moreover, pro-inflammatory signatures often correlate more strongly with active histological lesions, such as endocapillary hypercellularity and crescent formation, than with chronic fibrotic lesions. These observations suggest that tissue proteomics may distinguish biologically active inflammation from established irreversible damage, an important consideration for therapeutic decision-making.

Perhaps the greatest recent advance has been the emergence of spatial proteomics. Conventional tissue proteomics requires tissue homogenization, which loses information about the spatial distribution of proteins within the kidney. New spatially resolved methods, such as imaging mass spectrometry, digital spatial profiling, and highly multiplexed antibody-based imaging, not only preserve tissue architecture but also enable simultaneous quantification of hundreds of proteins across anatomically distinct regions. These techniques enable the depiction of molecular gradients in glomeruli, tubules, inflammatory cells, and fibrotic areas, thereby linking proteomic changes to cellular localization and histopathological context [57]. Although few cases of IgAN have been studied with this technology so far, spatial proteomics will likely be one of the key technologies transforming renal pathology over the next decade. Despite these advances, kidney tissue proteomics also has important limitations. Kidney biopsy is an invasive procedure performed primarily at diagnosis and is therefore unsuitable for repeated longitudinal assessment. The limited amount of available tissue restricts analytical depth, while intrarenal heterogeneity introduces sampling bias, particularly in focal diseases such as IgAN. Furthermore, current proteomic studies generally include relatively small patient cohorts due to limited availability of biopsy tissue, making independent validation difficult. Technical variability related to tissue processing, protein extraction, laser capture protocols, and mass spectrometry workflows also complicates comparisons between studies. Standardization of analytical pipelines remains essential before tissue proteomics can be incorporated into routine diagnostic pathology.

Nevertheless, kidney tissue proteomics serves as the biological reference against which plasma and urinary biomarkers should ultimately be interpreted. Plasma reflects systemic immune dysregulation, urine captures ongoing renal injury, whereas tissue proteomics directly identifies the molecular pathways operating within the diseased kidney. Integrating these complementary biological compartments offers an unprecedented opportunity to redefine IgAN in terms of molecular mechanisms rather than morphology alone. Future classification systems are therefore likely to combine histopathology with compartment-specific proteomic signatures to establish biologically meaningful molecular endotypes that improve prognostication and facilitate personalized therapeutic strategies.

### 3.4. Towards Molecular Endotypes Through Multi-Compartment Proteomics

**Figure 2:**
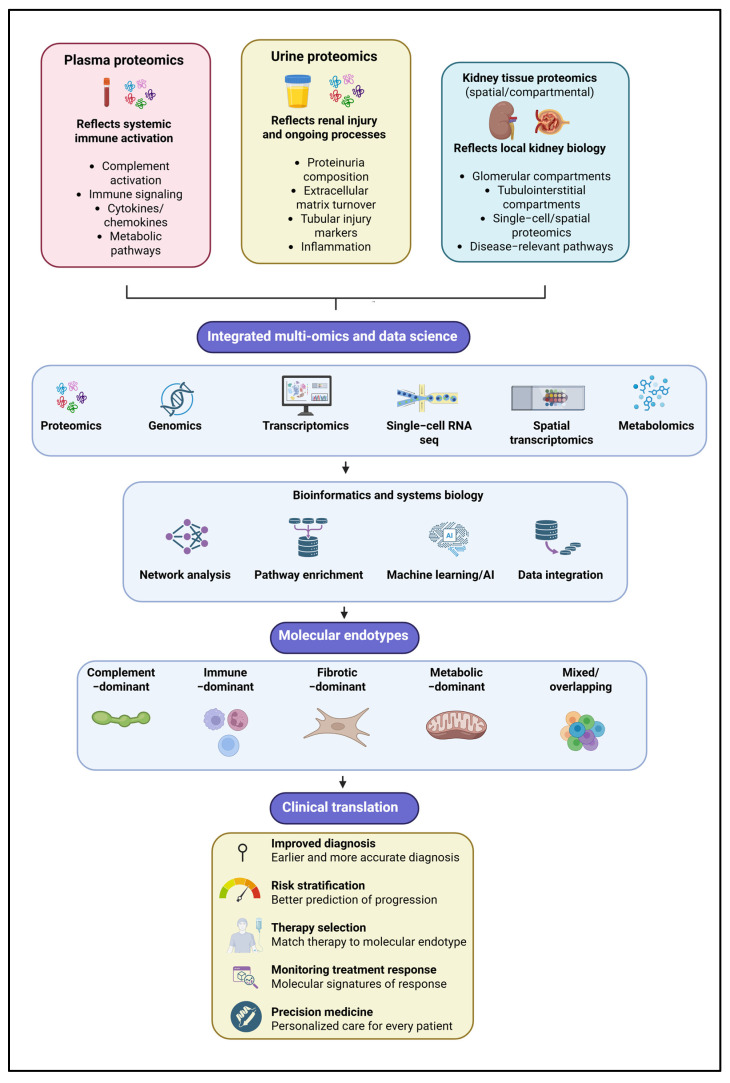
Multi-compartment proteomics integrates molecular information to define endotypes and enable precision nephrology in IgAN. The figure schematically represents how combining plasma, urinary, and kidney tissue proteomics can enhance the molecular characterization of IgAN. Plasma proteomics conceptually captures systemic immune dysregulation (including complement activation, inflammatory signaling pathways, and cytokine and chemokine networks) and metabolic changes. Urinary proteomics reflects a different aspect of ongoing intrarenal injury, driven by proteinuria, extracellular matrix remodeling, tubulointerstitial injury, and inflammation. Kidney tissue proteomics identifies local molecular processes in glomerular and tubulointerstitial compartments and can determine compartment-specific pathways using laser capture microdissection, high-resolution mass spectrometry, and spatial proteomic techniques. Combining these three biological compartments with genomics, transcriptomics, single-cell RNA sequencing, spatial transcriptomics, and metabolomics, and then applying systems biology and advanced bioinformatics, is the way to identify biologically meaningful molecular endotypes. These include complement-dominant, immune-dominant, fibrotic-dominant, metabolic-dominant, and mixed endotypes, which are believed to provide a level of biological detail far superior to that of traditional clinicopathological classification. In fact, this comprehensive multi-omics model might enable early diagnosis, enhanced risk stratification, selection of therapies based on mechanisms, longitudinal monitoring of treatment response, and the practice of precision nephrology in patients with IgAN. Abbreviations: AI, artificial intelligence; IgAN, IgA nephropathy; RNA, ribonucleic acid.

Although plasma, urinary, and kidney tissue proteomics each interrogate different biological compartments, the greatest value lies in their integration. Rather than providing isolated observations, these complementary datasets converge on a limited number of reproducible biological processes that collectively characterize the molecular heterogeneity of IgAN. Examining these findings together allows identification of biologically coherent molecular endotypes that may ultimately improve disease classification, prognostic stratification, and mechanism-based therapeutic selection. This integrated multi-compartment framework is summarized in Figure 2.

Across the three biological compartments, complement activation, immune dysregulation, extracellular matrix remodeling, and metabolic dysfunction are the most reproducible molecular processes. Although each compartment captures different aspects of disease biology, the convergence of these findings strongly supports biologically distinct, albeit partially overlapping, molecular endotypes. The principal molecular endotypes identified through integration of plasma, urinary, and kidney tissue proteomic studies, together with their dominant biological pathways, representative proteomic signatures, clinical associations, and potential therapeutic implications, are summarized in Table 3.

### 3.5. Integration of Plasma Proteomics

This concept is particularly important because IgAN is increasingly recognized as a biologically heterogeneous disease. The current Oxford MEST-C classification is an important tool for predicting patient outcomes by assessing structural damage. However, it does not identify the molecular processes underlying this damage. As a result, individuals with similar histopathological scores often show markedly different rates of disease progression and variable responses to immunosuppressive or targeted therapies [20,24]. These findings clearly indicate that identical histological phenotypes may arise from different underlying biological pathways.

Proteomics offers an opportunity to redefine this heterogeneity by identifying molecular endotypes rather than isolated biomarkers. Unlike conventional biomarkers, which quantify individual proteins associated with disease activity, molecular endotypes describe groups of patients who share common biological mechanisms. Such endotypes are expected to be biologically stable, mechanistically interpretable, and therapeutically actionable. Importantly, they are not defined by a single protein but by the coordinated activation of molecular pathways identified through systems biology approaches, including pathway enrichment analysis, protein–protein interaction networks, and unsupervised clustering [28].

Current evidence suggests that several reproducible molecular endotypes are emerging in IgAN. The most frequently observed feature of the condition is a complement-dominant endotype, which involves activation of the alternative and lectin complement pathways, impairment of the CFH-CFHR regulatory axis, and a build-up of complement proteins in plasma, urine, and glomerular tissue [5,36]. This endotype is supported by multiple lines of evidence from genome-wide association studies, plasma, urinary, and tissue proteomics, making it one of the strongest examples of biological consistency across multiple molecular compartments. The recent clinical success of complement inhibitors further underscores the therapeutic relevance of this molecular phenotype.

A second major endotype is characterized by immune activation and dysregulated mucosal immunity. Plasma proteomic studies consistently demonstrate activation of TNF-superfamily signaling, chemokine networks, B-cell differentiation, and plasma-cell biology, while transcriptomic studies have identified abnormalities in mucosal immune responses and immunoglobulin production [18,44]. This biological program directly relates to the production of galactose-deficient IgA1 and anti-glycan autoantibodies, the initiating events of the multi-hit hypothesis. The emergence of APRIL- and BAFF-targeted therapies highlights the clinical importance of identifying patients in whom this upstream immune program predominates.

A third molecular endotype features extracellular matrix remodeling and progressive fibrosis. Urinary peptide profiling and kidney tissue proteomics both reveal altered collagen turnover, elevated extracellular matrix protein levels, reduced matrix degradation, and activation of profibrotic pathways. Unlike inflammatory signatures, these changes appear to reflect permanent structural remodeling and may identify patients who are less likely to respond to immunomodulatory therapy but may be appropriate candidates for antifibrotic treatments. This distinction between inflammatory and fibrotic endotypes underscores the clinical utility of combining multiple proteomic compartments.

There is mounting evidence every day that a metabolic endotype exists, characterized by mitochondrial dysfunction, impaired oxidative phosphorylation, altered fatty acid metabolism, and cellular bioenergetic failure. Although complement or immune activation has been studied more extensively, analyses of the full set of proteins present in the kidney have repeatedly identified mitochondrial proteins as among the pathways most strongly linked to disease progression. These findings align with the growing recognition that metabolic dysfunction not only causes tubular injury and chronic kidney disease progression but also that inflammation is a secondary consequence.

The identification of these biological programs marks an important conceptual shift. Historically, biomarker discovery focused on identifying individual proteins with diagnostic or prognostic utility. However, the considerable biological complexity of IgAN makes it unlikely that any single circulating protein will capture the disease’s heterogeneity. Instead, integrating hundreds or thousands of proteins into coordinated molecular networks provides substantially greater biological resolution. Consequently, future research should prioritize systems biology approaches over traditional single-biomarker analyses.

Adding proteomics to other omics technologies advances our understanding in this area. A case in point is the use of genome-wide association studies to identify susceptibility genes related to antigen presentation, complement regulation, and mucosal immunity. Transcriptomic studies have also revealed inflammatory responses across various kidney cell types. Single-cell RNA sequencing has revealed remarkable heterogeneity among mesangial cells, podocytes, tubular epithelial cells, and immune cells, while spatial transcriptomics retains the geographical context of these molecular alterations. By aligning these supplementary datasets with proteomics through integrated analysis, we can now generate multi-layer molecular maps of IgAN that comprise genomic vulnerability, gene transcription, protein expression, and tissue distribution [25,58].

Artificial intelligence and cutting-edge computational biology could very well be at the heart of this transformation. Nowadays, machine-learning algorithms can integrate very high-dimensional proteomics, transcriptomics, genomics, and clinical datasets that are not accessible to traditional statistical methods. Methods such as network-based analyses, Bayesian modeling, or deep learning can reveal biologically relevant patient groups that are not identifiable through traditional clinico-pathological classification. Essentially, these computational methods should be used only as auxiliary tools for biological interpretation and not as replacements for it. This helps ensure that the identified phenotypes still make mechanistic sense and are viable in clinical settings.

However, there are still several roadblocks despite the huge capacity of molecular endotyping. For strong validation, large multicenter cohorts with standardized sample collection, harmonized analytical workflows, and comprehensive longitudinal follow-up are required. Also, integrating multiple proteomic platforms remains technically challenging, and we lack prospective interventional studies demonstrating improved patient outcomes with proteomic-guided treatment selection. Establishing standardized molecular definitions for proposed endotypes will therefore require close collaboration between nephrologists, molecular biologists, pathologists, bioinformaticians, and clinical trialists.

Nevertheless, the convergence of plasma, urinary, and tissue proteomics strongly suggests that IgAN should no longer be regarded as a single disease entity defined solely by histopathology. Instead, accumulating evidence supports a multidimensional classification in which systemic immune activation, complement dysregulation, extracellular matrix remodeling, and metabolic dysfunction define biologically distinct molecular endotypes. Such an approach has the potential to transform disease classification from descriptive morphology to mechanistic pathology and represents one of the most promising avenues for the development of precision nephrology.

## 4. Conclusions and Future Perspectives

The combined data from plasma, urine, and renal tissue proteomics suggest that information from these biological compartments does not compete but rather complements each other. Plasma proteomics mirrors whole-body immune dysregulation, complement activation, and inflammatory signaling before kidney injury occurs. Urine proteomics, by contrast, records the aftermath of these events, including glomerular barrier dysfunction, tubular injury, extracellular matrix remodeling, and disease progression. Renal tissue proteomics enables the direct identification of molecular changes specific to the compartments of the diseased kidney and provides the biological baseline for interpreting circulating and urinary markers. This approach provides a deeper understanding of IgAN than studying any of the three biological compartments in isolation and supports the development of a molecular disease classification.

Among the three methods, urinary proteomics is the one with the most clinical use these days. Large multicenter studies have validated urinary peptide biomarkers such as IgAN237 and shown that they are effective prognostic predictors across independent groups. By contrast, plasma proteomics is still largely in the discovery stage. Circulating proteomic studies have repeatedly identified alterations in several protein networks, including complement activation, immunity, metabolism, and the extracellular matrix. However, the number of plasma biomarkers that have been externally validated or evaluated prospectively in large multinational groups is very limited. Although kidney tissue proteomics reveals mechanisms uniquely, it remains mostly in preliminary form due to the lack of biopsy specimens, the difficulty of the procedure, and limited sample size.

Proteomics is also intended to complement genomic and transcriptomic methods rather than replace them. One of the functions of genomics is to reveal inherited predispositions, and, in a way, transcriptomics describes the activity of genes and cells. Proteomics, however, reveals operational molecular traits and records post-transcriptional regulation, protein–protein interactions, and post-translational modifications that one would only be aware of with the help of DNA or RNA. Because of this, for molecular subtyping and personalized kidney care, we will most probably resort to combined studies using many techniques, including genomics, transcriptomics, proteomics, and metabolomics, along with characterization and assessment through clinical phenotyping, rather than relying on a single one.

Despite that, the interpretation of the available data should still be done with great caution. There is major variability among studies in the selection of study participants, disease stages, patient race, data analysis methods, sample preparation, bioinformatic methods, outcome definitions, and follow-up duration. Most of these studies are still cross-sectional, have relatively small patient groups, and lack external validation. These methodological differences make it difficult to compare outcomes across studies and, as a result, limit the use of the proposed biomarkers in medical practice. Therefore, developing common analytical methods and reporting formats, and conducting large external validation studies, will be very important before proteomic biomarkers are adopted in routine clinical practice.

A realistic roadmap for clinical implementation should include the following sequential steps: (1) standardization of pre-analytical sample collection and proteomic workflows; (2) independent multicenter validation of candidate biomarker panels; (3) integration of plasma, urinary, and tissue proteomics with genomic, transcriptomic, and clinical data to define reproducible molecular endotypes; (4) prospective evaluation of proteomic classifiers in randomized clinical trials to establish prognostic and predictive utility; and (5) development of clinically applicable, cost-effective targeted assays suitable for routine diagnostic laboratories. Completing these steps would facilitate the transition of proteomics from biomarker discovery to precision nephrology, enabling mechanism-based patient stratification and individualized therapeutic selection in IgAN.

Overall, proteomics has revolutionized how we understand IgAN, a disease once recognized only through tissue biopsy pathology, and is now recognized as a disease with a range of molecular pathways and subtypes that reflect biological variation. Though plasma proteomics is not yet regularly used to inform clinical decisions, combining plasma, urine, and kidney tissue proteomics with other data, such as genomic, transcriptomic, and metabolomic information, presents an exciting direction for the field of nephrology toward a highly personalized medicine approach. Standardizing these methods to a higher degree, validating them across different centers, and conducting clinical follow-up studies will help determine whether proteomic endotyping will truly make a difference in predicting the future course of the disease, in selecting therapies based on mechanisms, and in ultimately saving the lives of IgAN patients better than before.

## Figures and Tables

**Table 1:** Comparison of proteomic platforms for plasma biomarker discovery in IgA nephropathy.

Platform	Proteome Coverage	Quantification	Strengths	Limitations	Best Application
Discovery LC-MS/MS (DDA)	High (2000 to 5000 proteins)	Relative	Unbiased discovery, identification of novel proteins	Missing values, lower reproducibility than DIA	Initial biomarker discovery
DIA-LC-MS/MS	Very high	Highly reproducible	Excellent quantitative reproducibility, deep proteome coverage	Requires spectral libraries and advanced bioinformatics	Large discovery cohorts, pathway analysis
Olink proximity extension assay	Targeted (up to approximately 5400 proteins depending on panel)	Relative	High sensitivity, excellent reproducibility, small sample volume	Limited to predefined proteins	Biomarker validation, epidemiology
SomaScan aptamer platform	Targeted (>7000 proteins)	Relative	Broad plasma coverage, high throughput	Aptamer specificity issues, orthogonal validation recommended	Large population studies
ELISA/targeted immunoassays	Single or few proteins	Absolute	Clinical applicability, inexpensive, regulatory familiarity	Cannot discover novel biomarkers	Clinical validation and implementation

Abbreviations: DDA, data-dependent acquisition; DIA, data-independent acquisition; ELISA, enzyme-linked immunosorbent assay; LC-MS/MS, liquid chromatography–tandem mass spectrometry; MALDI-TOF MS, matrix-assisted laser desorption/ionization time-of-flight mass spectrometry; Olink PEA, Olink proximity extension assay; SomaScan, slow off-rate modified aptamer scan; MS, mass spectrometry.

**Table 2:** Representative plasma proteomic discovery studies in IgA nephropathy.

Study	Cohort Size	Proteomic Platform	Key Findings
Early gel-based studies	Small discovery cohorts (generally < 50 patients)	Two-dimensional gel electrophoresis (2-DE)	First demonstration that the circulating proteome differs between patients with IgAN and healthy controls; identification of acute-phase, complement, coagulation, and lipid metabolism proteins.
Early SELDI-TOF/MS studies	Small discovery cohorts	Surface-enhanced laser desorption/ionization time-of-flight mass spectrometry (SELDI-TOF-MS)	Identification of disease-associated plasma protein fingerprints and proof-of-concept for plasma proteomic profiling.
Early MALDI-TOF studies	Small discovery cohorts	Matrix-assisted laser desorption/ionization time-of-flight mass spectrometry (MALDI-TOF MS)	Identification of discriminatory peptide signatures associated with IgAN.
Zhang et al. [27]	IgAN patients and healthy controls	LC-MS/MS combined with metabolomics	Demonstrated coordinated alterations in complement activation, immune signaling, amino acid metabolism, and energy metabolism, highlighting pathway-level dysregulation rather than individual biomarkers.
Chen et al. [9]	IgAN cohort	DIA-LC-MS/MS	Identified molecular proteomic subtypes associated with clinical outcome, supporting the concept of molecular endotypes.
Recent affinity-based studies	Large validation cohorts	Olink proximity extension assay (PEA) and SomaScan	High-throughput validation of inflammatory and immune-related protein signatures and translation of candidate biomarkers into larger clinical studies.

Abbreviations: 2-DE, two-dimensional gel electrophoresis; DIA, data-independent acquisition; DIA-LC-MS/MS, data-independent acquisition liquid chromatography–tandem mass spectrometry; IgAN, IgA nephropathy; LC-MS/MS, liquid chromatography–tandem mass spectrometry; MALDI-TOF MS, matrix-assisted laser desorption/ionization time-of-flight mass spectrometry; PEA, proximity extension assay; SELDI-TOF-MS, surface-enhanced laser desorption/ionization time-of-flight mass spectrometry.

**Table 3:** Proposed molecular endotypes in IgA nephropathy based on integrated plasma, urinary, and kidney tissue proteomics.

Molecular Endotype	Dominant Molecular Pathways	Primary Supporting Proteomic Evidence	Potential Clinical Implications	Representative Targeted Therapies
Complement-dominant	Alternative and lectin complement pathway activation (CFH/CFHR axis, C3, properdin, MASP-2)	Primarily plasma proteomics, supported by urinary and kidney tissue proteomics	Patients with predominant complement activation may benefit from complement-targeted therapies	Factor B inhibitors (iptacopan), Factor D inhibitors (danicopan), MASP-2 inhibitors (narsoplimab), C3 inhibitors (pegcetacoplan)
Immune-inflammatory	BAFF/APRIL signaling, TNF receptor pathways, chemokines, cytokines, B-cell and plasma-cell activation	Primarily plasma proteomics, supported by urinary and kidney tissue proteomics	Identification of patients with dominant immune activation who may respond to immunomodulatory therapies	APRIL inhibitors (sibeprenlimab, zigakibart), BAFF/APRIL inhibitors (atacicept, telitacicept, povetacicept), targeted-release budesonide
Fibrotic/remodeling	Extracellular matrix remodeling, collagen turnover, TGF-β signaling, tubulointerstitial fibrosis	Predominantly kidney tissue and urinary proteomics; currently limited direct plasma proteomic evidence	May identify patients with advanced chronic remodeling and irreversible kidney injury	Antifibrotic approaches and supportive therapies (investigational)
Metabolic	Mitochondrial dysfunction, oxidative stress, altered energy metabolism, amino acid metabolism	Predominantly kidney tissue and urinary proteomics with supportive plasma metabolomic/proteomic studies	May identify patients with metabolic reprogramming and progressive disease	Metabolism-targeted therapies (investigational)
Endotype overlap	Simultaneous activation of multiple pathogenic pathways	Evidence suggests substantial overlap between molecular pathways rather than mutually exclusive endotypes	Combined pathway activation may ultimately require individualized combination therapies	To be determined

Abbreviations: APRIL, A Proliferation-Inducing Ligand; BAFF, B-cell Activating Factor; C3, Complement component 3; CFH, Complement Factor H; CFHR, Complement Factor H-Related protein; MASP-2, Mannan-binding lectin-associated serine protease 2; TNF, Tumor Necrosis Factor; TGF-β, Transforming Growth Factor-beta.

## Data Availability

No new data were created or analyzed in this study.

## Abbreviations

The following abbreviations are used in this manuscript:

AI	Artificial intelligence
APRIL	A Proliferation-Inducing Ligand
AUC	Area under the curve
BAFF	B-cell Activating Factor
BAFF-R	B-cell Activating Factor Receptor
BCMA	B-cell Maturation Antigen
C	Cellular/Fibrocellular Crescents (Oxford Classification)
C1q	Complement component 1q
C3	Complement component 3
C4d	Complement component 4d
C5a	Complement component 5a
C5b-9	Terminal complement complex (membrane attack complex)
CCL2	C-C motif chemokine ligand 2
CFH	Complement factor H
CFHR	Complement factor H-related protein
CFHR1	Complement factor H-related protein 1
CFHR5	Complement factor H-related protein 5
CKD	Chronic kidney disease
CXCL1	C-X-C motif chemokine ligand 1
CXCL12	C-X-C motif chemokine ligand 12
DDA	Data-dependent acquisition
DIA	Data-independent acquisition
E	Endocapillary hypercellularity (Oxford Classification)
eGFR	Estimated glomerular filtration rate
ELISA	Enzyme-linked immunosorbent assay
GALT	Gut-associated lymphoid tissue
Gd-IgA1	Galactose-deficient immunoglobulin A1
GWAS	Genome-wide association study
IgA	Immunoglobulin A
IgA1	Immunoglobulin A1
IgAN	IgA nephropathy
IgG	Immunoglobulin G
IL	Interleukin
IL-6	Interleukin-6
IL6ST	Interleukin-6 signal transducer (gp130)
LC-MS/MS	Liquid chromatography–tandem mass spectrometry
M	Mesangial hypercellularity (Oxford Classification)
MALDI-TOF	Matrix-assisted laser desorption/ionization time-of-flight
MALDI-TOF MS	Matrix-assisted laser desorption/ionization time-of-flight mass spectrometry
MASP-2	Mannose-binding lectin-associated serine protease 2
MCP-1	Monocyte chemoattractant protein-1
MEST-C	Mesangial hypercellularity, Endocapillary hypercellularity, Segmental glomerulosclerosis, Tubular atrophy/interstitial fibrosis, and Cellular/Fibrocellular Crescents
MS	Mass spectrometry
Olink PEA	Olink Proximity Extension Assay
PEA	Proximity Extension Assay
PRKAR2A	Protein kinase cAMP-dependent type II regulatory subunit alpha
RNA	Ribonucleic acid
SELDI-TOF-MS	Surface-enhanced laser desorption/ionization time-of-flight mass spectrometry
S	Segmental glomerulosclerosis (Oxford Classification)
SOS1	Son of Sevenless homolog 1
SGLT2	Sodium-glucose cotransporter 2
T	Tubular atrophy/interstitial fibrosis (Oxford Classification)
TACI	Transmembrane Activator and CAML Interactor
TGF-β	Transforming growth factor-beta
TNF-α	Tumor necrosis factor-alpha
TNFR	Tumor necrosis factor receptor
TNFR1	Tumor necrosis factor receptor 1
TNFR2	Tumor necrosis factor receptor 2
TNFSF10	Tumor necrosis factor ligand superfamily member 10 (TRAIL)
TNFSF12	Tumor necrosis factor ligand superfamily member 12 (TWEAK)
TRAIL	TNF-related apoptosis-inducing ligand
TWEAK	TNF-like weak inducer of apoptosis
